# Preventing Pandemics Via International Development: A Systems Approach

**DOI:** 10.1371/journal.pmed.1001354

**Published:** 2012-12-11

**Authors:** Tiffany L. Bogich, Rumi Chunara, David Scales, Emily Chan, Laura C. Pinheiro, Aleksei A. Chmura, Dennis Carroll, Peter Daszak, John S. Brownstein

**Affiliations:** 1 Fogarty International Center, National Institutes of Health, Bethesda, Maryland, United States of America; 2 Princeton University, Dept of Ecology & Evolutionary Biology, Princeton, New Jersey, United States of America; 3 EcoHealth Alliance, New York, New York, United States of America; 4 Children's Hospital Informatics Program, Division of Emergency Medicine, Children's Hospital Boston, Boston, Massachusetts, United States of America; 5 Harvard Medical School, Department of Pediatrics, Boston, Massachusetts, United States of America; 6 Global Health Program, United States Agency for International Development (USAID), Washington (D.C.), United States of America

## Abstract

Tiffany Bogich and colleagues find that breakdown or absence of public health infrastructure is most often the driver in pandemic outbreaks, whose prevention requires mainstream development funding rather than emergency funding.

**Summary Points**

- The way in which public health programs are designed and funded has changed significantly; however, the trend toward establishing vertical, disease-specific global health programs may be at the cost of strengthening basic public health infrastructure and development in the long term.
- In a review of nearly 400 public health events of international concern, we found that a breakdown or absence of public health infrastructure was the driving factor in the largest fraction of outbreaks (39.5%). No single other driving factor accounted for more than 10% of outbreaks.
- The relative roles of emergency response versus long-term development strategies to mitigate infectious disease threats are being debated within bilateral and intergovernmental aid agencies.
- We propose a systems approach within development agencies to address pandemic prevention at the intersection of people and their environment where the risk of disease emergence is highest. To achieve this goal, mainstream development funding, rather than emergency funding, is required.

## Outbreaks, Driving Factors, and Development

**Figure 1:**
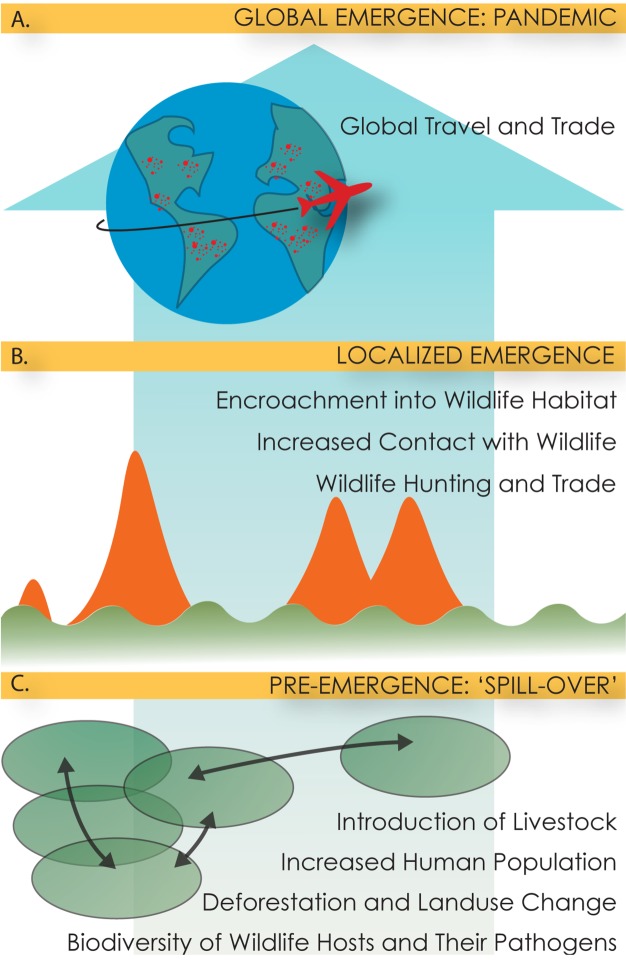
Figurative description of the multi-scale, multi-step process of pandemic emergence. Pandemic impact is highest when diseases are transmitted rapidly from human to human, and spread via travel and trade networks (A). At that point, their impact is greatest in developed countries, with economic dependence on globalized travel and trade (e.g., SARS). However, most emerging diseases do not reach this stage, and emerge in localized outbreaks, often small and contained (B, red spikes), or spillover repeatedly from animals (B, green line). Here, control is most effective at the countries of origins that are often developing countries, where breakdown of public health measures exacerbates human-to-human spread. Prior to localized outbreaks of zoonoses, perturbations in the environment lead to spillover of pathogens from one animal species to another or their range expansion (C, green circles). The most effective pandemic prevention at this early stage would be via measures that target the underlying causes of disease emergence.

Outbreaks of emerging infectious diseases place significant burden on public health and global economies [1]. The process leading to spillover, localized emergence, and finally pandemic spread is complex, but is generally driven by underlying ecological, political, or socioeconomic changes [2],[3] (Figure 1). These “drivers” [2],[4] include for example, climate change, urbanization, international travel and trade, land use change, and the breakdown or complete lack of public health measures. Yet despite the growing literature on driving factors [4], the impact of these drivers lacks appropriate attention and is currently an understudied area of research [5].

Examining a dataset of outbreaks of international concern assembled as part of a recently published study by Chan et al. [6], we assess the distribution of outbreaks across driving factors. We find that the most prominent driver is the breakdown or lack of public health infrastructure and argue that there is a mismatch between the drivers of public health events and current trends in public health spending and pandemic prevention. We propose a three-pronged approach within development agencies as the most promising solution to this disconnect. The approach includes: (1) Developing policies that deal with different stages of emergence, from spillover and localized outbreaks to pandemic spread; (2) Actively engaging a systems approach to pandemic prevention that changes pathogen dynamics at the intersection of people and their environment; and (3) Shifting the funding framework in development agencies from short-term emergency funding to a longer-term strategy.

## Determining Drivers of Outbreaks of International Concern

Epidemiological data on officially confirmed outbreaks of international concern collected by the Global Alert and Response (GAR) department of the World Health Organization (WHO) can facilitate understanding of threats to global health, with particular attention to the local spread of pathogens (Figure 1B) at the critical juncture following spillover into humans but preceding pandemic spread. Events of “international concern” are published in Disease Outbreak News (DON, available online, http://www.who.int/csr/don/en/) and defined according to the International Health Regulations (IHR) as either specific diseases (Table S1) or events that are “serious” or “unusual” or pose the potential risk of spreading globally or imposing restrictions on travel or trade (http://www.who.int/ihr). The inclusion criteria for events evolved from 1969 when the IHR covered only six diseases, to amendments in 1973 and 1982 to focus specifically on cholera, yellow fever, and plague, and to revisions again in 1995 to cover almost all public health risks (biological, chemical, radiological, or nuclear), though these final revisions were only formally adopted in 2005 and did not go into effect until 15 June 2007. The IHR now require states to have or develop “minimum core public health capacities,” including the detection, assessment, and notification of events.

**Figure 2:**
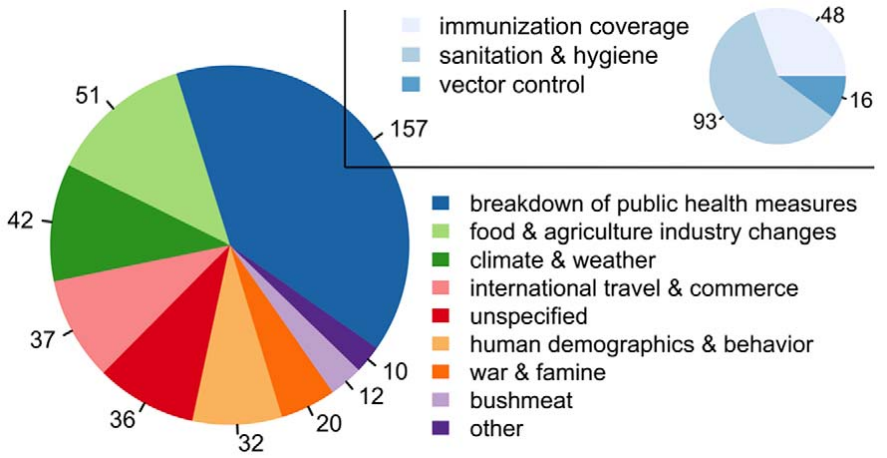
The number of outbreaks by driver, with the subplot representing the subdrivers within the category “breakdown of public health measures” (Table S3). The number of outbreaks as taken from the Chan et al. [6] dataset of DON reports attributed to different ecological, socioeconomic, and political drivers. There are some inherent uncertainties in the reported set of outbreaks, and biases in the reporting of disease outbreaks have been discussed previously [4],[6]. As the breakdown of public health measures accounted for the greatest number of outbreaks, for that driver, three additional subcategories were examined, including inadequate sanitation and hygiene, poor immunization coverage, and vector-borne and zoonotic disease control measures (e.g., bednets and improved drainage to eliminate standing water). Drivers associated with fewer than ten events (which included “human susceptibility to infection,” “land use changes,” and “medical industry changes”) were combined into the single category “other.” Outbreak events with unassigned or uncertain drivers (e.g., disagreement between sources) were labeled as “unspecified.”

Using the Chan et al. [6] dataset of 397 outbreaks from DON reports between 1996 and 2009, we identified the proximate driver implicated in each outbreak through manual evaluation of WHO outbreak reports related to each event (Text S1). Driving factors were defined according to the Institute of Medicine (IOM) [2] with modifications as in Jones et al. [4], including the re-classification of “economic development and land use” and “technology and industry” to form more descriptive categories: “agricultural industry changes,” “medical industry changes,” “food industry changes,” “land use changes,” and “bushmeat” (See Text S1 for a full list of drivers). Drivers were assigned on the basis of a text search of the outbreak reports for key words and phrases indicating an IOM-defined driver directly or inferred from text describing actions taken immediately following the outbreak (Table S2). Breakdown of public health measures accounted for the largest fraction (39.5%) of outbreaks (Figure 2; Table S3). According to the IOM [2], breakdown of public health measures includes inadequate sanitation and hygiene, e.g., the shortage of potable water, poor immunization coverage or the lack of infrastructure to purchase and deliver vaccine, and the deterioration of vector-borne and zoonotic disease control. We include the absence of public health infrastructure in “breakdown of public health measures,” but use the IOM naming construct of “breakdown” for consistency. All other drivers accounted for 10% or less of outbreaks each (Table S3). While many of these outbreaks do not pose a pandemic threat, they are evidence of an environment that may prove unable to deal with a novel pandemic threat.

## Limitations to Determining Drivers of Outbreaks of International Concern

The ability to accurately assess the driving factors of outbreaks and target aid is limited by strong disincentives that still exist for states to report outbreaks of disease (e.g., disruption to tourism or trade) [7]. Further, a lack of standard practices for sharing biological samples limits our ability to detect and report disease threats rapidly. The Nagoya Protocol to the Convention on Biological Diversity set the groundwork for sample sharing, but does not include human samples and still requires significant deliberation prior to implementation of a fair sharing system [7].

Despite disincentives and surveillance issues, the significant subset of events that do get reported thorough IHR mechanisms point to a role for development in preventing future outbreaks. Currently, the role of international development in global health and pandemic prevention programs in the United States and internationally is being debated.

## The Changing Role of International Development in Pandemic Prevention

Historically, dealing with infectious disease outbreak threats has been under the aegis of state, national, and international public health agencies (e.g., US Centers for Disease Control, WHO), and basic public health infrastructural development has been the responsibility of bilateral and international development aid agencies (e.g., United States Agency for International Development [USAID] and World Bank, respectively), and national and international non-governmental organizations (e.g., Bill & Melinda Gates Foundation). There has been an increased role for bilateral and international development aid agencies in addressing pandemic prevention as a development issue, typically funded through emergency response avenues. This shift followed the emergence of highly pathogenic H5N1 avian influenza, in which developing countries such as Indonesia and Egypt were identified as regions with repeated, small-scale outbreaks that suggested chronic persistence [8]. The connection between H5N1 and backyard poultry production in particular has led to significant interest and support in a “systems approach” to combating avian influenza as a development agenda driven by agricultural, cultural, poverty, and equity constraints, rather than a purely human health issue (also see http://www.apeiresearch.net/main.php) [9]. These efforts have led to broader programs including inter-agency “One Health” initiatives, and global programs specifically targeting pandemic infectious disease threats through development [10],[11].

Within development agencies trends in health spending have moved from broad systems based investments to a more specialized, infectious disease model, resulting in a decline in systems capacities, potentially contributing to increased risk of disease spread. According to the Organization for Economic Co-operation and Development (OECD), infectious disease control aid commitments have increased from 8% between 1990 and 1998 to 16% between 2005 and 2008, while basic health infrastructure aid commitments have declined from 11% to 5% during this period. In response, aid has been criticized as duplicative and inefficient, aimed at high-profile diseases (e.g., HIV/AIDS) rather than public health in general, and too often tied to short-term numerical targets, such as patients treated or lives saved [12]. Further, the proliferation of donors with program-specific “earmarking” of funds for vertical spending may create a fragmented landscape of development aid and translate into additional costs on donor and recipient countries [13]. Therefore, the decline in broad health systems capacities could be due not simply to the structure of aid being too vertical or “stovepiped” along “high profile diseases,” but also to inflexible funding cycles bent on metrics with little long-term effect. While vertical programs do have their successes, often programmatic and structural details helped overcome the vertical nature of the program, e.g., the high coverage of excellent and evolving vertically oriented interventions that contributed to smallpox eradication [14].

## Conclusion and Recommendations

We suggest a central role for development agencies in pandemic prevention and highlight three critical policy issues. The first is to develop policies that deal with different stages of emergence, from spillover and localized outbreaks to pandemic spread. Stronger public health infrastructure (e.g., expanded surveillance, better diagnostic capacity, and rapid reporting and control) in developing countries will likely help prevent localized outbreaks of newly emerged pathogens becoming pandemic. For example, in China the SARS crisis exposed weaknesses in the health system and the ability to effectively communicate and control an epidemic threat [15]. China has made a series of changes to public health policy and infrastructure specifically targeting SARS-like illnesses, as well as other emerging diseases [16],[17]. It is likely that any future spillover of SARS, either from animals or via laboratory accidents, or emergence of a similar but novel disease would be less likely to result in international spread [17]–[19]. Similarly, extensive national and intergovernmental efforts to detect and control influenza A/H5N1 in Indonesia and other southeast Asian countries may have played a role in the lack of sustained human-to-human transmission in the region [20]. The majority of the events that the WHO has classified as internationally significant are in fact vaccine preventable or can be contained with basic public health measures, e.g., yellow fever, polio, cholera, and meningitis, making the bulk of events in the Chan et al. [6] dataset preventable. These generalized approaches are distinct from efforts to target specific diseases that have emerged, particularly those with rapid, silent (during the asymptomatic period) transmission, such as the proposed distribution of oseltamivir as a prophylaxis during the early stages of the 2009 H1N1 pandemic [21]. Here, the practicalities of distribution among individuals or households to achieve optimal coverage proved difficult and this model of pandemic control via prophylaxis is seen as overly optimistic [22],[23], especially in the context of a developing country.

Second, we propose that development agencies should actively engage a systems approach to pandemic prevention that changes pathogen dynamics at the intersection of people and their environment, broadening the development toolkit significantly and imaginatively. A systems approach to pandemic prevention moves beyond the “One Health” concept of linking human and veterinary medicine with an understanding of environmental drivers of health to focus also on the socio-ecological context of disease emergence [24]. There has been significant movement in One Health, however, “operationalizing” One Health seems to hit a glass ceiling because there is not a specific defined budget among the agencies, and each relevant agency competes for funds. For H5N1, reducing the risk of the emergence of a new pandemic variant includes increasing biosecurity on poultry farms and within backyard flocks [25] as well as strengthening surveillance along routes from farms to markets. To address the key drivers of most pandemics, this will mean development agencies playing a role in such diverse strategies as strengthening animal health diagnostic laboratories, training veterinarians in public health (e.g., epidemiology for disease surveillance, outbreak detection, investigation, and intervention), the promotion of biosecurity measures on farms, educating bushmeat hunters on disease risks, and working with the extractive industries in emerging infectious disease “hotspots” to reduce the risk of new pathogens emerging (See figure 1 in [6]). In 2009, USAID launched the Emerging Pandemic Threats program with the specific aim of reducing opportunity for the emergence of new, potentially pandemic zoonoses at their source by building local public health capacity to predict, identify, respond to, and prevent disease emergence (http://avianflu.aed.org/eptprogram/).

Third and finally, we point to the need for a critical shift in the funding framework from which disease-oriented development assistance is administered. Within development agencies, pandemic prevention programs are most commonly funded through emergency or disaster relief mechanisms. In this transition, development agencies should consider adopting a long-term funding strategy that invests in a development approach to pandemic prevention consistent with a systems approach. These recommendations align with others who have urged previously for a strengthening of national health systems with a “diagonal” approach [26], where interest in particular initiatives or diseases can be used to drive broad-based improvements to the overall public health system. Not only will this better address the most significant global health threats, but it will also provide the broad scale first line of defense against the next unknown contagion.

## Supporting Information

Table S1 **WHO Diseases of Focus (http://www.who.int/csr/disease/en/).**
(PDF)

Table S2 **Summary of outbreak information extracted from the WHO Disease Outbreak News reports by disease, country, and date, with corresponding driver classification.**
(PDF)

Table S3 **Number of outbreaks by driver as used in** **Figure 2** **.**
(PDF)

Text S1 **Supplemental online material.**
(DOC)

## Abbreviations

IHR: International Health Regulations
WHO: World Health Organization
IOM: Institute of Medicine
