# Supplementary material for: Preventing Pandemics Via International Development: A Systems Approach
Source: PLoS Med. 2012 Dec 11;9(12):e1001354. doi: 10.1371/journal.pmed.1001354 (PMC3519898; doi:10.1371/journal.pmed.1001354)
Supplement: Table S1 — WHO Diseases of Focus (http://www.who.int/csr/disease/en/). (PDF) [file pmed.1001354.s001.pdf]

Supplementary Table S1: WHO Diseases of Focus (http://www.who.int/csr/disease/en/)
Note, the WHO reports have also reported on other diseases such as:
cholera, poliomyelitis, Legionnaires', typhoid and shigellosis

**Disease**
Anthrax
Avian influenza
Crimean-Congo haemorrhagic fever (CCHF)
Dengue/dengue haemorrhagic fever
Ebola haemorrhagic fever
Hendra Virus (HeV) Infection
Hepatitis
Influenza
Lassa fever
Marburg haemorrhagic fever
Meningococcal disease
Human Monkeypox (MPX)
Nipah Virus (NiV) Infection
Pandemic (H1N1) 2009
Plague
Rift Valley fever
Severe Acute Respiratory Syndrome (SARS)
Smallpox
Tularaemia
Yellow fever
