# Supplementary material for: Preventing Pandemics Via International Development: A Systems Approach
Source: PLoS Med. 2012 Dec 11;9(12):e1001354. doi: 10.1371/journal.pmed.1001354 (PMC3519898; doi:10.1371/journal.pmed.1001354)
Supplement: Table S2 — Summary of outbreak information extracted from the WHO Disease Outbreak News reports by disease, country, and date, with corresponding driver classification. (PDF) [file pmed.1001354.s002.pdf]

Supplementary Table S2: Summary of outbreak information extracted from the WHO Disease Outbreak News Reports by disease, country and date, with corresponding driver classificatio

Disease	Country	Date	General Driver	Driver Details	Link
Malaria	Burundi	25 June 1999	breakdown of public health measures	vector control	http://www.who.int/csr/don/1999_06_25/en/
Measles	Ireland	09 August 2000	breakdown of public health measures	immunization coverage	http://www.who.int/csr/don/2000_08_09/en/
Yellow Fever	Benin	02 October 1996	breakdown of public health measures	vector control	http://www.who.int/csr/don/1996_10_02/en/
Measles	South Korea	09 February 2001	breakdown of public health measures	immunization coverage	http://www.who.int/csr/don/2001_02_09/en/
Yellow Fever	Brazil	05 February 2008	breakdown of public health measures	immunization coverage	http://www.who.int/csr/don/2008_02_07/en/
Poliomyelitis	Albania	18 September 1996	breakdown of public health measures	immunization coverage	http://www.who.int/csr/don/1996_09_18/en/
Yellow Fever	Bolivia	11 February 1999	breakdown of public health measures	vector control	http://www.who.int/csr/don/1999_02_11/en/
Yellow Fever	Brazil	26 August 1998	breakdown of public health measures	vector control	http://www.who.int/csr/don/1998_08_26/en/
Yellow Fever	Burkina Faso	22 December 1998	breakdown of public health measures	vector control	http://www.who.int/csr/don/1998_12_22a/en/
Yellow Fever	Colombia	18 May 1999	breakdown of public health measures	vector control	http://www.who.int/csr/don/1999_05_18a/en/
Yellow Fever	Cote d'Ivoire	04 September 2001	breakdown of public health measures	vector control	http://www.who.int/csr/don/2001_09_04/en/
Yellow Fever	Cote d'Ivoire	31 May 2001	breakdown of public health measures	vector control	http://www.who.int/csr/don/2001_05_31/en/
Yellow Fever	Ghana	05 December 1996	breakdown of public health measures	vector control	http://www.who.int/csr/don/1996_12_05/en/
Yellow Fever	Guinea	25 September 2001	breakdown of public health measures	vector control	http://www.who.int/csr/don/2001_09_25a/en/
Yellow Fever	Peru	18 May 1999	breakdown of public health measures	vector control	http://www.who.int/csr/don/1999_05_18a/en/
Yellow Fever	Senegal	21 January 2002	breakdown of public health measures	vector control	http://www.who.int/csr/don/2002_01_21/en/
Yellow Fever	Sudan	27 May 2003	breakdown of public health measures	immunization coverage	http://www.who.int/csr/don/2003_05_27/en/
Poliomyelitis	Somalia	13 September 2005	breakdown of public health measures	immunization coverage	http://www.who.int/csr/don/2005_09_13/en/
Measles	Nigeria	17 March 2005	breakdown of public health measures	immunization coverage	http://www.who.int/csr/don/2005_03_17/en/
Yellow Fever	Burkina Faso	03 November 2008	breakdown of public health measures	immunization coverage	http://www.who.int/csr/don/2008_11_03/en/
Yellow Fever	Burkina Faso	24 November 2004	breakdown of public health measures	immunization coverage	http://www.who.int/csr/don/2004_11_24/en/
Yellow Fever	Central African Republic	01 December 2009	breakdown of public health measures	immunization coverage	http://www.who.int/csr/don/2009_12_01/en/
Yellow Fever	Central African Republic	20 May 2008	breakdown of public health measures	immunization coverage	http://www.who.int/csr/don/2008_05_20/en/
Yellow Fever	Cote d'Ivoire	08 August 2008	breakdown of public health measures	immunization coverage	http://www.who.int/csr/don/2008_08_08/en/index.html
Yellow Fever	Cote d'Ivoire	19 October 2006	breakdown of public health measures	immunization coverage	http://www.who.int/csr/don/2006_10_19a/en/
Yellow Fever	Guinea	02 November 2005	breakdown of public health measures	immunization coverage	http://www.who.int/csr/don/2005_11_02/en/
Yellow Fever	Guinea	14 January 2009	breakdown of public health measures	immunization coverage	http://www.who.int/csr/don/2009_01_14/en/
Yellow Fever	Guinea	29 September 2008	breakdown of public health measures	immunization coverage	http://www.who.int/csr/don/2008_09_29/en/
Yellow Fever	Liberia	18 April 2008	breakdown of public health measures	immunization coverage	http://www.who.int/csr/don/2008_04_18/en/
Yellow Fever	Mali	03 November 2005	breakdown of public health measures	immunization coverage	http://www.who.int/csr/don/2005_11_03/en/
Yellow Fever	Paraguay	20 February 2008	breakdown of public health measures	immunization coverage	http://www.who.int/csr/don/2008_02_20a/en/
Yellow Fever	Senegal	20 October 2005	breakdown of public health measures	immunization coverage	http://www.who.int/csr/don/2005_10_20/en/
Yellow Fever	Sierra Leone	06 January 2009	breakdown of public health measures	immunization coverage	http://www.who.int/csr/don/2009_01_06a/en/index.html
Yellow Fever	Sudan	21 November 2005	breakdown of public health measures	immunization coverage	http://www.who.int/csr/don/2005_11_21/en/
Yellow Fever	Togo	19 December 2006	breakdown of public health measures	immunization coverage	http://www.who.int/csr/don/2006_12_19/en/
Poliomyelitis	Nigeria; Benin; Niger (West/Central Africa)	18 June 2008	breakdown of public health measures	immunization coverage	http://www.who.int/csr/don/2008_06_18/en/
Measles	Guadeloupe	17 January 1997	breakdown of public health measures	immunization coverage	http://www.who.int/csr/don/1997_01_17a/en/
Meningitis	Central African Republic	16 February 2000	breakdown of public health measures	immunization coverage	http://www.who.int/csr/don/2000_02_16/en/
Meningitis	DR Congo	10 October 2001	breakdown of public health measures	immunization coverage	http://www.who.int/csr/don/2001_10_10b/en/index.html
Meningitis	Ethiopia	17 March 2000	breakdown of public health measures	immunization coverage	http://www.who.int/csr/don/2000_03_17/en/
Meningitis	Rwanda	20 October 1999	breakdown of public health measures	immunization coverage	http://www.who.int/csr/don/1999_10_20/en/
Yellow Fever	Brazil	17 January 2003	breakdown of public health measures	immunization coverage	http://www.who.int/csr/don/2003_01_17/en/
Yellow Fever	Brazil	17 March 1999	breakdown of public health measures	immunization coverage	http://www.who.int/csr/don/1999_03_17b/en/

Disease	Country	Date	General Driver	Driver Details	Link
Yellow Fever	Brazil	28 February 2001	breakdown of public health measures	immunization coverage	http://www.who.int/csr/don/2001_02_28a/en/
Yellow Fever	Burkina Faso	10 October 2003	breakdown of public health measures	immunization coverage	http://www.who.int/csr/don/2003_10_10/en/
Yellow Fever	Burkina Faso	11 May 2004	breakdown of public health measures	immunization coverage	http://www.who.int/csr/don/2004_05_11/en/
Yellow Fever	Guinea	03 February 2003	breakdown of public health measures	immunization coverage	http://www.who.int/csr/don/2003_02_03/en/
Yellow Fever	Guinea	07 January 2005	breakdown of public health measures	immunization coverage	http://www.who.int/csr/don/2005_01_07/en/index.html
Yellow Fever	Guinea	29 December 2000	breakdown of public health measures	immunization coverage	http://www.who.int/csr/don/2000_12_29/en/
Yellow Fever	Guinea	31 August 2005	breakdown of public health measures	immunization coverage	http://www.who.int/csr/don/2005_08_31/en/
Yellow Fever	Liberia	23 August 2001	breakdown of public health measures	immunization coverage	http://www.who.int/csr/don/2001_08_23/en/
Yellow Fever	Liberia	24 August 2000	breakdown of public health measures	immunization coverage	http://www.who.int/csr/don/2000_08_24b/en/
Yellow Fever	Nigeria	19 May 2000	breakdown of public health measures	immunization coverage	http://www.who.int/csr/don/2000_05_19/en/index.html
Yellow Fever	Peru	07 June 2001	breakdown of public health measures	immunization coverage	http://www.who.int/csr/don/2001_06_07e/en/
Yellow Fever	Senegal	04 October 2002	breakdown of public health measures	immunization coverage	http://www.who.int/csr/don/2002_10_04/en/
Yellow Fever	Sierra Leone	19 August 2003	breakdown of public health measures	immunization coverage	http://www.who.int/csr/don/2003_08_19/en/
Yellow Fever	Venezuela	04 November 1998	breakdown of public health measures	immunization coverage	http://www.who.int/csr/don/1998_11_04/en/
Yellow Fever	Venezuela	14 September 2004	breakdown of public health measures	immunization coverage	http://www.who.int/csr/don/2004_09_14/en/
Cholera	Burundi	18 February 2004	breakdown of public health measures	sanitation & hygiene	http://www.who.int/csr/don/2004_02_18a/en/
Cholera	Chad	26 July 2001	breakdown of public health measures	sanitation & hygiene	http://www.who.int/csr/don/2001_07_26a/en/
Cholera	Cote d'Ivoire	08 January 2003	breakdown of public health measures	sanitation & hygiene	http://www.who.int/csr/don/2003_01_08/en/index.html
Cholera	DR Congo	16 September 2002	breakdown of public health measures	sanitation & hygiene	http://www.who.int/csr/don/2002_09_16/en/
Cholera	Guinea	26 September 2001	breakdown of public health measures	sanitation & hygiene	http://www.who.int/csr/don/2001_09_26/en/
Cholera	Iraq	08 May 2003	breakdown of public health measures	sanitation & hygiene	http://www.who.int/csr/don/2003_05_08a/en/index.html
Cholera	Liberia	03 July 2003	breakdown of public health measures	sanitation & hygiene	http://www.who.int/csr/don/2003_07_03a/en/
Cholera	Liberia	13 September 2002	breakdown of public health measures	sanitation & hygiene	http://www.who.int/csr/don/2002_09_13a/en/
Cholera	Malawi	26 March 2002	breakdown of public health measures	sanitation & hygiene	http://www.who.int/csr/don/2002_03_26/en/
Cholera	Mozambique	21 May 2003	breakdown of public health measures	sanitation & hygiene	http://www.who.int/csr/don/2003_05_21/en/
Cholera	Mozambique	28 January 2004	breakdown of public health measures	sanitation & hygiene	http://www.who.int/csr/don/2004_01_28/en/
Cholera	Tanzania	26 July 2001	breakdown of public health measures	sanitation & hygiene	http://www.who.int/csr/don/2001_07_26/en/
Cholera	Zambia	09 May 2003	breakdown of public health measures	sanitation & hygiene	http://www.who.int/csr/don/2003_05_09/en/index.html
Cholera	Comoros Islands	22 December 1999	breakdown of public health measures	sanitation & hygiene	http://www.who.int/csr/don/1999_12_22/en/
Dysentery	Sierra Leone	24 January 2000	breakdown of public health measures	sanitation & hygiene	http://www.who.int/csr/don/2000_01_24a/en/
Cholera	Cote d'Ivoire	13 August 2002	breakdown of public health measures	sanitation & hygiene	http://www.who.int/csr/don/2002_08_13a/en/index.html
Cholera	DR Congo	15 November 2002	breakdown of public health measures	sanitation & hygiene	http://www.who.int/csr/don/2002_11_15/en/
Cholera	Senegal	02 November 2004	breakdown of public health measures	sanitation & hygiene	http://www.who.int/csr/don/2004_11_02/en/
Cholera	Sierra Leone	14 September 1999	breakdown of public health measures	sanitation & hygiene	http://www.who.int/csr/don/1999_09_14/en/
Plague	DR Congo	13 October 2006	breakdown of public health measures	sanitation & hygiene	http://www.who.int/csr/don/2006_10_13/en/
Plague	Malawi	27 July 1999	breakdown of public health measures	sanitation & hygiene	http://www.who.int/csr/don/1999_07_27/en/
Cholera	Brazil	08 April 1999	breakdown of public health measures	sanitation & hygiene	http://www.who.int/csr/don/1999_04_08/en/
Cholera	Burundi	28 May 1998	breakdown of public health measures	sanitation & hygiene	http://www.who.int/csr/don/1998_05_28/en/
Cholera	Chile	12 January 1998	breakdown of public health measures	sanitation & hygiene	http://www.who.int/csr/don/1998_01_12a/en/
Cholera	Iraq	26 October 1998	breakdown of public health measures	sanitation & hygiene	http://www.who.int/csr/don/1998_10_26/en/
Cholera	Madagascar	14 April 1999	breakdown of public health measures	sanitation & hygiene	http://www.who.int/csr/don/1999_04_14a/en/
Cholera	Mali	25 November 2003	breakdown of public health measures	sanitation & hygiene	http://www.who.int/csr/don/2003_11_25/en/
Cholera	Micronesia	06 July 2000	breakdown of public health measures	sanitation & hygiene	http://www.who.int/csr/don/2000_07_06/en/
Cholera	Mozambique	28 June 2002	breakdown of public health measures	sanitation & hygiene	http://www.who.int/csr/don/2002_06_28/en/
Cholera	Sierra Leone	23 September 1998	breakdown of public health measures	sanitation & hygiene	http://www.who.int/csr/don/1998_09_23/en/
Cholera	Somalia	18 December 1998	breakdown of public health measures	sanitation & hygiene	http://www.who.int/csr/don/1998_12_18/en/
Cholera	Somalia	22 November 2000	breakdown of public health measures	sanitation & hygiene	http://www.who.int/csr/don/2000_11_22/en/

Disease	Country	Date	General Driver	Driver Details	Link
Cholera	South Africa	13 October 2000	breakdown of public health measures	sanitation & hygiene	http://www.who.int/csr/don/2000_10_13/en/
Cholera	Sri Lanka	25 November 1998	breakdown of public health measures	sanitation & hygiene	http://www.who.int/csr/don/1998_11_25/en/
Cholera	Sudan	24 February 2006	breakdown of public health measures	sanitation & hygiene	http://www.who.int/csr/don/2006_02_24/en/
Hand Foot and Mouth Disease	China	01 May 2008	breakdown of public health measures	sanitation & hygiene	http://www.who.int/csr/don/2008_05_01/en/
Hand Foot and Mouth Disease	Malaysia	06 June 1997	breakdown of public health measures	sanitation & hygiene	http://www.who.int/csr/don/1997_07_03/en/
Plague	Malawi	05 June 2002	breakdown of public health measures	sanitation & hygiene	http://www.who.int/csr/don/2002_06_05e/en/index.html
Cholera	Angola	10 May 2006	breakdown of public health measures	sanitation & hygiene	http://www.who.int/csr/don/2006_05_10/en/
Cholera	Armenia	11 September 1998	breakdown of public health measures	sanitation & hygiene	http://www.who.int/csr/don/1998_09_11/en/
Cholera	Benin	20 November 2003	breakdown of public health measures	sanitation & hygiene	http://www.who.int/csr/don/2003_11_20/en/
Cholera	Bhutan	31 August 1998	breakdown of public health measures	sanitation & hygiene	http://www.who.int/csr/don/1998_08_31/en/
Cholera	Bolivia	22 January 1997	breakdown of public health measures	sanitation & hygiene	http://www.who.int/csr/don/1997_01_22/en/
Cholera	Burkina Faso	17 September 2001	breakdown of public health measures	sanitation & hygiene	http://www.who.int/csr/don/2001_09_17a/en/
Cholera	Burundi	09 January 1997	breakdown of public health measures	sanitation & hygiene	http://www.who.int/csr/don/1997_01_09a/en/
Cholera	Cambodia	15 July 1998	breakdown of public health measures	sanitation & hygiene	http://www.who.int/csr/don/1998_07_15/en/
Cholera	Cameroon	15 October 1998	breakdown of public health measures	sanitation & hygiene	http://www.who.int/csr/don/1998_10_15/en/
Cholera	Cameroon	18 February 2004	breakdown of public health measures	sanitation & hygiene	http://www.who.int/csr/don/2004_02_18a/en/
Cholera	Central African Republic	30 July 1997	breakdown of public health measures	sanitation & hygiene	http://www.who.int/csr/don/1997_07_30/en/
Cholera	Chad	05 July 1996	breakdown of public health measures	sanitation & hygiene	http://www.who.int/csr/don/1996_07_05/en/
Cholera	Comoros Islands	10 February 1998	breakdown of public health measures	sanitation & hygiene	http://www.who.int/csr/don/1998_02_10b/en/
Cholera	Congo Republic	03 February 1998	breakdown of public health measures	sanitation & hygiene	http://www.who.int/csr/don/1998_02_03a/en/index.html
Cholera	Cote d'Ivoire	17 September 2001	breakdown of public health measures	sanitation & hygiene	http://www.who.int/csr/don/2001_09_17a/en/index.html
Cholera	DR Congo	05 March 2002	breakdown of public health measures	sanitation & hygiene	http://www.who.int/csr/don/2002_03_05/en/
Cholera	Iraq	10 September 2007	breakdown of public health measures	sanitation & hygiene	http://www.who.int/csr/don/2007_09_10a/en/
Cholera	Kenya	20 January 1999	breakdown of public health measures	sanitation & hygiene	http://www.who.int/csr/don/1999_01_20/en/
Cholera	Kenya	22 September 1997	breakdown of public health measures	sanitation & hygiene	http://www.who.int/csr/don/1997_09_22/en/
Cholera	Mongolia	13 August 1996	breakdown of public health measures	sanitation & hygiene	http://www.who.int/csr/don/1996_08_13b/en/
Cholera	Nicaragua; Honduras	23 June 1999	breakdown of public health measures	sanitation & hygiene	http://www.who.int/csr/don/1999_06_23/en/
Cholera	Niger	17 September 2001	breakdown of public health measures	sanitation & hygiene	http://www.who.int/csr/don/2001_09_17a/en/
Cholera	Niger	24 July 2002	breakdown of public health measures	sanitation & hygiene	http://www.who.int/csr/don/2002_07_24/en/
Cholera	Nigeria	01 March 1996	breakdown of public health measures	sanitation & hygiene	http://www.who.int/csr/don/1996_03_01a/en/
Cholera	Nigeria	27 November 2001	breakdown of public health measures	sanitation & hygiene	http://www.who.int/csr/don/2001_11_27/en/
Cholera	Philippines	12 September 1996	breakdown of public health measures	sanitation & hygiene	http://www.who.int/csr/don/1996_09_12/en/
Cholera	Philippines	14 June 1996	breakdown of public health measures	sanitation & hygiene	http://www.who.int/csr/don/1996_06_14c/en/
Cholera	Tanzania	06 May 1997	breakdown of public health measures	sanitation & hygiene	http://www.who.int/csr/don/1997_05_06b/en/
Cholera	Uganda	07 April 1998	breakdown of public health measures	sanitation & hygiene	http://www.who.int/csr/don/1998_04_07/en/
Cholera	Uganda	19 May 2003	breakdown of public health measures	sanitation & hygiene	http://www.who.int/csr/don/1998_04_07/en/
Cholera	Zimbabwe	07 April 1998	breakdown of public health measures	sanitation & hygiene	http://www.who.int/csr/don/1998_04_07b/en/
Plague	Namibia	11 May 1999	bushmeat		http://www.who.int/csr/don/1999_05_11/en/
Ebola	Congo Republic	13 May 2005	bushmeat		http://www.who.int/csr/don/2005_05_13/en/index.html
Ebola	DR Congo	26 December 2008	bushmeat		http://www.who.int/csr/don/2008_12_26a/en/
Ebola	DR Congo	31 August 2007	bushmeat		http://www.who.int/csr/don/2007_08_31a/en/index.html
Ebola	Gabon	14 October 1996	bushmeat		http://www.who.int/csr/don/1996_10_14/en/
Ebola	Gabon	19 February 1996	bushmeat		http://www.who.int/csr/don/1996_02_19b/en/
Ebola	Sudan	18 May 2004	bushmeat		http://www.who.int/csr/don/2004_05_18/en/index.html

Disease	Country	Date	General Driver	Driver Details	Link
Ebola	Uganda	16 October 2000	bushmeat		http://www.who.int/csr/don/2000_10_16/en/
Ebola	Uganda	30 November 2007	bushmeat		http://www.who.int/csr/don/2007_11_30a/en/
Monkeypox	DR Congo	21 March 1997	bushmeat		http://www.who.int/csr/don/1997_03_21c/en/
Plague	Malawi	30 October 1997	climate & weather		http://www.who.int/csr/don/1997_10_30/en/
Plague	Mozambique	08 August 1997	climate & weather		http://www.who.int/csr/don/1997_08_08/en/
Cholera	Djibouti	21 November 1997	climate & weather		http://www.who.int/csr/don/1997_11_21b/en/
Cholera	Mozambique	02 September 1997	climate & weather		http://www.who.int/csr/don/1997_09_02b/en/
Cholera	Zambia	28 January 2004	climate & weather		http://www.who.int/csr/don/2004_01_28/en/
Dengue	Venezuela	13 August 1996	climate & weather		http://www.who.int/csr/don/1996_08_13a/en/
Dengue	Brazil	21 March 2002	climate & weather		http://www.who.int/csr/don/2002_03_21/en/
Dengue	Cook Islands	28 February 1997	climate & weather		http://www.who.int/csr/don/1997_02_28a/en/
Dengue	El Salvador	21 June 2002	climate & weather		http://www.who.int/csr/don/2002_06_21e/en/
Dengue	India	17 October 1996	climate & weather		http://www.who.int/csr/don/1996_10_17a/en/
Dengue	Indonesia	26 February 2004	climate & weather		http://www.who.int/csr/don/2004_02_26a/en/
Dengue	Madagascar; Maldives	17 March 2006	climate & weather		http://www.who.int/csr/don/2006_03_17/en/
Dengue	Timor-Leste	31 January 2005	climate & weather		http://www.who.int/csr/don/2005_01_31/en/
Encephalitis	United States	10 September 1999	climate & weather		http://www.who.int/csr/don/1999_09_10/en/
West Nile Virus	Canada	12 September 2002	climate & weather		http://www.who.int/csr/don/2002_09_12b/en/
West Nile Virus	United States	27 August 2002	climate & weather		http://www.who.int/csr/don/2002_08_27/en/
Avian Influenza	China	04 December 2007	food & agriculture industry changes		http://www.who.int/csr/don/2007_12_04/en/index.html
Avian Influenza	China	20 February 2008	food & agriculture industry changes		http://www.who.int/csr/don/2008_02_20/en/index.html
Avian Influenza	Indonesia	21 July 2005	food & agriculture industry changes		http://www.who.int/csr/don/2005_07_21a/en/
Avian Influenza	Nigeria	31 January 2007	food & agriculture industry changes		http://www.who.int/csr/don/2007_01_31a/en/
Avian Influenza	Vietnam	13 January 2004	food & agriculture industry changes		http://www.who.int/csr/don/2004_01_13/en/
Avian Influenza	Hong Kong	05 January 1998	food & agriculture industry changes		http://www.who.int/csr/don/1998_01_05/en/index.html
Avian Influenza	Hong Kong	07 April 1999	food & agriculture industry changes		http://www.who.int/csr/don/1999_04_07c/en/index.html
Avian Influenza	Hong Kong	19 February 2003	food & agriculture industry changes		http://www.who.int/csr/don/2003_2_19/en/
Hantavirus	Panama	02 March 2000	food & agriculture industry changes		http://www.who.int/csr/don/2000_03_02a/en/
Japanese Encephalitis	Nepal	04 October 1996	food & agriculture industry changes		http://www.who.int/csr/don/1996_10_04/en/
Marburg Fever	DR Congo	30 April 1999	food & agriculture industry changes		http://www.who.int/csr/don/1999_04_30/en/
Plague	Zambia	20 March 2001	food & agriculture industry changes		http://www.who.int/csr/don/2001_03_20a/en/
Swine Influenza (H1N1)	Mexico	24 April 2009	food & agriculture industry changes		http://www.who.int/csr/don/2009_04_24/en/index.html
Typhoid	Tadjikistan	28 February 1997	food & agriculture industry changes		http://www.who.int/csr/don/1997_02_28b/en/
Plague	Algeria	24 June 2003	food & agriculture industry changes		http://www.who.int/csr/don/2003_06_24a/en/
Leptospirosis	Kenya	17 June 2004	human demographics & behavior		http://www.who.int/csr/don/2004_06_17a/en/
Plague	DR Congo	14 June 2006	human demographics & behavior		http://www.who.int/csr/don/2006_06_14/en/
Chikungunya	France	17 February 2006	human demographics & behavior		http://www.who.int/csr/don/2006_02_17a/en/
Meningitis	Mozambique	11 July 1996	human demographics & behavior		http://www.who.int/csr/don/1996_07_11/en/
Cholera	Niger	03 August 1999	human demographics & behavior		http://www.who.int/csr/don/1999_08_03/en/
Diphtheria	Laos; Thailand	19 July 1996	human demographics & behavior		http://www.who.int/csr/don/1996_07_19a/en/
Influenza	DR Congo	29 November 2002	human demographics & behavior		http://www.who.int/csr/don/2002_11_29a/en/index.html
Leptospirosis	India	27 October 1997	human demographics & behavior		http://www.who.int/csr/don/1997_10_27/en/
Meningitis	Angola	06 August 1998	human demographics & behavior		http://www.who.int/csr/don/1998_08_06/en/
Meningitis	Angola	30 July 1996	human demographics & behavior		http://www.who.int/csr/don/1996_07_30b/en/
Meningitis	Cyprus	12 August 1996	human demographics & behavior		http://www.who.int/csr/don/1996_08_12b/en/

Disease	Country	Date	General Driver	Driver Details	Link
Meningitis	Guinea-Bissau	26 February 1999	human demographics & behavior		http://www.who.int/csr/don/1999_02_26b/en/
Meningitis	Hungary	01 January 2000	human demographics & behavior		http://www.who.int/csr/don/2000_01_01/en/index.html
Meningitis	Romania	02 September 1996	human demographics & behavior		http://www.who.int/csr/don/1996_09_02a/en/
Meningitis	Somalia	07 December 2001	human demographics & behavior		http://www.who.int/csr/don/2001_12_07/en/index.html
Plague	India	20 February 2002	human demographics & behavior		http://www.who.int/csr/don/2002_02_20/en/
Shigellosis	Cameroon	29 April 1998	human demographics & behavior		http://www.who.int/csr/don/1998_04_29/en/
Undiagnosed Pneumonia	Sudan	22 October 1998	human demographics & behavior		http://www.who.int/csr/don/1998_10_22b/en/
Whooping Cough	Afghanistan	08 January 2003	human demographics & behavior		http://www.who.int/csr/don/2003_01_08a/en/
Enterovirus	Taiwan	17 June 1998	human demographics & behavior		http://www.who.int/csr/don/1998_06_17e/en/
Meningitis	Palestinian Authority	07 July 1997	human demographics & behavior		http://www.who.int/csr/don/1997_07_07/en/
Meningitis	Uganda	25 January 2006	human susceptibility to infection		http://www.who.int/csr/don/2006_01_25/en/index.html
Meningitis	Sudan	12 January 1999	human susceptibility to infection		http://www.who.int/csr/don/1999_01_12/en/
Lassa Fever	Sierra Leone	15 May 1996	international travel & commerce		http://www.who.int/csr/don/1996_05_15/en/
Avian Influenza	Thailand	23 January 2004	international travel & commerce		http://www.who.int/csr/don/2004_01_23/en/
Cholera	South Africa	18 February 2004	international travel & commerce		http://www.who.int/csr/don/2004_02_18a/en/
Dengue	Honduras	19 July 2002	international travel & commerce		http://www.who.int/csr/don/2002_07_19b/en/
Influenza	DR Congo	12 February 2003	international travel & commerce		http://www.who.int/csr/don/2003_02_12/en/
Lassa Fever	Sierra Leone	20 April 2004	international travel & commerce		http://www.who.int/csr/don/2004_04_20a/en/
Meningitis	Rwanda	24 August 2000	international travel & commerce		http://www.who.int/csr/don/2000_08_24/en/index.html
Nipah/Hendra Virus	Bangladesh	12 February 2004	international travel & commerce		http://www.who.int/csr/don/2004_02_12/en/
Nipah/Hendra Virus	Bangladesh	20 April 2004	international travel & commerce		http://www.who.int/csr/don/2004_04_20/en/
Nipah/Hendra Virus	Bangladesh	25 May 2001	international travel & commerce		http://www.who.int/csr/don/2001_05_25a/en/index.html
[illegible] Virus	Malaysia; Singapore	22 March 1999	international travel & commerce		http://www.who.int/csr/don/1999_03_22a/en/index.html
Poliomyelitis	Angola	28 April 1999	international travel & commerce		http://www.who.int/csr/don/1999_04_28b/en/
Poliomyelitis	Chad	29 October 2003	international travel & commerce		http://www.who.int/csr/don/2003_10_29/en/
Poliomyelitis	Yemen	25 April 2005	international travel & commerce		http://www.who.int/csr/don/2005_04_25/en/
SARS	China	11 February 2003	international travel & commerce		http://www.who.int/csr/don/2003_02_11/en/index.html
West Nile Virus	Israel	22 September 2000	international travel & commerce		http://www.who.int/csr/don/2000_09_22/en/index.html
Meningitis	Kenya	03 March 2006	international travel & commerce		http://www.who.int/csr/don/2006_03_03a/en/
Avian Influenza	Cambodia	02 February 2005	international travel & commerce		http://www.who.int/csr/don/2005_02_02/en/
Meningitis	Burundi	23 August 2002	international travel & commerce		http://www.who.int/csr/don/2002_08_23a/en/index.html
Meningitis	Rwanda	23 August 2002	international travel & commerce		http://www.who.int/csr/don/2002_08_23a/en/index.html
Rift Valley Fever	Madagascar	18 April 2008	land use changes		http://www.who.int/csr/don/2008_04_18a/en/
Rift Valley Fever	Saudi Arabia	18 September 2000	land use changes		http://www.who.int/csr/don/2000_09_18/en/
Rift Valley Fever	Sudan	05 November 2007	land use changes		http://www.who.int/csr/don/2007_11_05/en/
Rift Valley Fever	Tanzania	23 March 2007	land use changes		http://www.who.int/csr/don/2007_03_23/en/
Cholera	Brunei Darussalam	23 June 1999	unspecified		http://www.who.int/csr/don/1999_06_23/en/
Influenza	Madagascar	05 August 2002	unspecified		http://www.who.int/csr/don/2002_08_05/en/
Meningitis	Angola	15 August 2001	unspecified		http://www.who.int/csr/don/2001_08_15a/en/index.html
Meningitis	Central African Republic	08 April 2004	unspecified		http://www.who.int/csr/don/2004_04_08a/en/index.html
Meningitis	Chad	08 April 2004	unspecified		http://www.who.int/csr/don/2004_04_08b/en/index.html
Meningitis	Ethiopia	19 March 1999	unspecified		http://www.who.int/csr/don/1999_03_19b/en/index.html

Disease	Country	Date	General Driver	Driver Details	Link
Meningitis	Zimbabwe	02 September 1997	unspecified		http://www.who.int/csr/don/1997_09_02a/en/
O'nyong-Nyong Fever	Uganda	12 March 1997	unspecified		http://www.who.int/csr/don/1997_03_12/en/
Shigellosis	Guinea	14 July 1999	unspecified		http://www.who.int/csr/don/1999_07_14/en/
Shigellosis	Liberia	30 September 2003	unspecified		http://www.who.int/csr/don/2003_09_30/en/index.html
Typhoid	Kyrgyzstan	21 September 1998	unspecified		http://www.who.int/csr/don/1998_09_21/en/
Dengue	Cuba	18 June 1997	unspecified		http://www.who.int/csr/don/1997_06_18/en/
Cholera	Sudan	26 March 1999	unspecified		http://www.who.int/csr/don/1999_03_26a/en/index.html
Avian Influenza	Egypt	26 December 2007	unspecified		http://www.who.int/csr/don/2007_12_26a/en/index.html
Avian Influenza	Vietnam	28 December 2007	unspecified		http://www.who.int/csr/don/2007_12_28/en/
Dengue	Ecuador	16 August 2002	unspecified		http://www.who.int/csr/don/2002_08_16/en/
Dengue	Ecuador	16 August 2002	unspecified		http://www.who.int/csr/don/2002_08_16/en/
Marburg Fever	Uganda	03 August 2007	unspecified		http://www.who.int/csr/don/2007_08_03/en/
Meningitis	India	09 May 2005	unspecified		http://www.who.int/csr/don/2005_05_09/en/index.html
Meningitis	Philippines	11 January 2005	unspecified		http://www.who.int/csr/don/2005_01_11/en/
Meningitis	Sudan	03 February 2006	unspecified		http://www.who.int/csr/don/2006_02_03/en/index.html
Myocarditis	Sri Lanka	17 March 2005	unspecified		http://www.who.int/csr/don/2005_03_17a/en/
Influenza	Afghanistan	24 February 1999	unspecified		http://www.who.int/csr/don/1999_02_24/en/index.html
Relapsing Fever	Sudan	07 April 1999	unspecified		http://www.who.int/csr/don/1999_04_07b/en/index.html
Plague	Uganda	06 November 1998	unspecified		http://www.who.int/csr/don/1998_11_06a/en/
Marburg Fever	Angola	17 March 2005	war & famine		http://www.who.int/csr/don/2005_03_17b/en/index.html
Crimean-Congo Hemorrhagic Fever	Mauritania	11 March 2003	war & famine		http://www.who.int/csr/don/2003_03_11/en/
Dengue	Cambodia; southeast Asia (Malaysia; Taiwan; Cambodia; Vietnam; Thailand; Philippines; Indonesia; Myanmar)	13 February 1998	war & famine		http://www.who.int/csr/don/1998_02_13/en/index.html
Dengue	India	30 October 2003	war & famine		http://www.who.int/csr/don/2003_10_30/en/
Dengue	Malaysia	31 July 1997	war & famine		http://www.who.int/csr/don/1997_07_31a/en/
Undiagnosed Hemorrhagic Fever	Afghanistan	19 June 2000	war & famine		http://www.who.int/csr/don/2000_06_19e/en/
Cholera	Niger	29 July 2005	war & famine		http://www.who.int/csr/don/2005_07_29b/en/
Typhoid	Algeria	22 January 1996	breakdown of public health measures	sanitation & hygiene	http://www.who.int/csr/don/1996_01_22e/en/
Cholera	Chad	28 January 2004	breakdown of public health measures	sanitation & hygiene	http://www.who.int/csr/don/2004_01_28/en/
Legionnaires'	Australia	04 May 2000	breakdown of public health measures	sanitation & hygiene	http://www.who.int/csr/don/2000_05_04/en/
Legionnaires'	Spain	12 July 2001	breakdown of public health measures	sanitation & hygiene	http://www.who.int/csr/don/2001_07_12/en/index.html
Cholera	Brazil	25 November 1998	breakdown of public health measures	sanitation & hygiene	http://www.who.int/csr/don/1998_11_25a/en/
Cholera	Burundi	19 July 2002	breakdown of public health measures	sanitation & hygiene	http://www.who.int/csr/don/2002_07_19/en/
Cholera	Nigeria	12 May 1999	breakdown of public health measures	sanitation & hygiene	http://www.who.int/csr/don/1999_05_12/en/
Cholera	Nigeria	03 December 2004	breakdown of public health measures	sanitation & hygiene	http://www.who.int/csr/don/2004_12_03/en/
Cholera	Russia	23 October 1998	breakdown of public health measures	sanitation & hygiene	http://www.who.int/csr/don/1998_10_23/en/
Cholera	Rwanda	18 November 1999	breakdown of public health measures	sanitation & hygiene	http://www.who.int/csr/don/1999_11_18/en/
Cholera	Zimbabwe	02 December 2008	breakdown of public health measures	sanitation & hygiene	http://www.who.int/csr/don/2008_12_02/en/
Typhoid	DR Congo	15 December 2004	breakdown of public health measures	sanitation & hygiene	http://www.who.int/csr/don/2004_12_15/en/

Disease	Country	Date	General Driver	Driver Details	Link
Hepatitis	Sudan	10 August 2004	breakdown of public health measures	sanitation & hygiene	http://www.who.int/csr/don/2004_08_10/en/
Cholera	Cambodia	21 May 1999	breakdown of public health measures	sanitation & hygiene	http://www.who.int/csr/don/1999_05_21/en/
Cholera	DR Congo	06 January 1998	breakdown of public health measures	sanitation & hygiene	http://www.who.int/csr/don/1998_01_06a/en/
Cholera	Iraq	10 September 2008	breakdown of public health measures	sanitation & hygiene	http://www.who.int/csr/don/2008_09_10a/en/
Dysentery	Lesotho	28 January 2000	breakdown of public health measures	sanitation & hygiene	http://www.who.int/csr/don/2000_01_28a/en/
Cholera	Somalia	02 May 2000	climate & weather		http://www.who.int/csr/don/2000_05_02/en/
Cholera	Somalia	15 April 1997	climate & weather		http://www.who.int/csr/don/1997_04_15a/en/index.html
Malaria	Kenya	14 July 1999	climate & weather		http://www.who.int/csr/don/1999_07_14a/en/
Cholera	Burundi	23 June 1999	climate & weather		http://www.who.int/csr/don/1999_06_23/en/
Cholera	Bolivia	31 March 1998	climate & weather		http://www.who.int/csr/don/1998_03_31/en/
Cholera	Ecuador	31 March 1998	climate & weather		http://www.who.int/csr/don/1998_03_31/en/
Cholera	Honduras	31 March 1998	climate & weather		http://www.who.int/csr/don/1998_03_31/en/
Cholera	Nicaragua	31 March 1998	climate & weather		http://www.who.int/csr/don/1998_03_31/en/
Cholera	Ethiopia	04 October 2006	climate & weather		http://www.who.int/csr/don/2006_10_04/en/index.html
Cholera	Chad	01 September 2004	climate & weather		http://www.who.int/csr/don/2004_09_01/en/
Cholera	Peru	25 February 1998	climate & weather		http://www.who.int/csr/don/1998_02_25/en/
Cholera	Liberia	03 August 1998	climate & weather		http://www.who.int/csr/don/1998_08_03/en/
Cholera	Niger	02 June 2004	climate & weather		www.who.int/csr/don/2004_06_02/en/
Malaria	Tanzania	19 May 1998	climate & weather		http://www.who.int/csr/don/1998_05_19/en/index.html
Cholera	Mozambique	03 February 1998	climate & weather		http://www.who.int/csr/don/1998_02_03b/en/
Cholera	DR Congo	09 December 1999	climate & weather		http://www.who.int/csr/don/1999_12_09/en/
Cholera	India	14 August 2001	climate & weather		http://www.who.int/csr/don/2001_08_14/en/
Typhoid	Haiti	17 June 2003	climate & weather		http://www.who.int/csr/don/2003_06_17a/en/
Avian Influenza	Cambodia	24 March 2006	food & agriculture industry changes		http://www.who.int/csr/don/2006_03_24/en/
Avian Influenza	Turkey	05 January 2006	food & agriculture industry changes		http://www.who.int/csr/don/2006_01_18/en/
Streptococcus suis	China	03 August 2005	food & agriculture industry changes		http://www.who.int/csr/don/2005_08_03/en/
Avian Influenza	Canada	05 April 2004	food & agriculture industry changes		http://www.who.int/csr/don/2004_04_05/en/
Avian Influenza	China	01 March 2007	food & agriculture industry changes		http://www.who.int/csr/don/2007_03_01a/en/
Avian Influenza	China	07 January 2009	food & agriculture industry changes		http://www.who.int/csr/don/2009_01_07a/en/index.html
Avian Influenza	Egypt	16 December 2008	food & agriculture industry changes		http://www.who.int/csr/don/2008_12_16/en/
Avian Influenza	Egypt	20 March 2006	food & agriculture industry changes		http://www.who.int/csr/don/2006_03_20/en/
Avian Influenza	Iraq	30 January 2006	food & agriculture industry changes		http://www.who.int/csr/don/2006_01_30a/en/
Avian Influenza	Laos	27 February 2007	food & agriculture industry changes		http://www.who.int/csr/don/2007_02_27/en/
Avian Influenza	Pakistan	15 December 2007	food & agriculture industry changes		http://www.who.int/csr/don/2007_12_15/en/
Avian Influenza	Thailand	09 September 2004	food & agriculture industry changes		http://www.who.int/csr/don/2004_09_09/en/
Avian Influenza	Thailand	20 October 2005	food & agriculture industry changes		http://www.who.int/csr/don/2005_10_20a/en/
Avian Influenza	Thailand	26 July 2006	food & agriculture industry changes		http://www.who.int/csr/don/2006_07_26/en/
Avian Influenza	United Kingdom	29 May 2007	food & agriculture industry changes		http://www.who.int/csr/don/2007_05_29/en/
Avian Influenza	Vietnam	12 August 2004	food & agriculture industry changes		http://www.who.int/csr/don/2004_08_12/en/
Avian Influenza	Vietnam	09 November 2005	food & agriculture industry changes		http://www.who.int/csr/don/2005_11_09/en/
Avian Influenza	Vietnam	07 January 2009	food & agriculture industry changes		http://www.who.int/csr/don/2009_01_07/en/
Avian Influenza	Vietnam	29 June 2007	food & agriculture industry changes		http://www.who.int/csr/don/2007_06_29/en/
Anthrax	Ethiopia	20 June 2000	food & agriculture industry changes		http://www.who.int/csr/don/2000_07_20/en/
Avian Influenza	Netherlands	24 April 2003	food & agriculture industry changes		http://www.who.int/csr/don/2003_04_24/en/
Japanese Encephalitis	Malaysia	22 March 1999	food & agriculture industry changes		http://www.who.int/csr/don/1999_03_22a/en/

Disease	Country	Date	General Driver	Driver Details	Link
Avian Influenza	Vietnam	30 December 2004	food & agriculture industry changes		http://www.who.int/csr/don/2004_12_30/en/
Avian Influenza	Egypt	27 December 2006	food & agriculture industry changes		http://www.who.int/csr/don/2006_12_27a/en/
Plague	China	11 August 2009	food & agriculture industry changes		http://www.who.int/csr/don/2009_08_11/en/
Avian Influenza	Azerbaijan	10 March 2006	food & agriculture industry changes		http://www.who.int/csr/don/2006_03_10a/en/
Anthrax	Ghana	05 June 1997	food & agriculture industry changes		http://www.who.int/csr/don/1997_06_05/en/
Meningitis	Uganda	24 January 2007	human demographics & behavior		http://www.who.int/csr/don/2007_01_24/en/
Yellow Fever	[illegible] d'Ivoire	22 September 2005	human demographics & behavior		http://www.who.int/csr/don/2005_09_22/en/
Plague	DR Congo	18 February 2005	human demographics & behavior		http://www.who.int/csr/don/2005_02_18/en/
Leptospirosis	France	15 September 2000	international travel & commerce		http://www.who.int/csr/don/2000_09_21/en/index.html
Meningitis	United Kingdom; Spain	31 May 1996	international travel & commerce		http://www.who.int/csr/don/1996_05_31/en/
Cholera	South Africa	23 May 2003	international travel & commerce		http://www.who.int/csr/don/2003_05_23a/en/
Legionnaires'	France	14 August 2003	international travel & commerce		http://www.who.int/csr/don/2003_08_14a/en/index.html
Poliomyelitis	West Africa (Ghana; Togo; Burkina Faso)	09 October 2003	international travel & commerce		http://www.who.int/csr/don/2003_10_09/en/index.html
Dengue	Cape Verde	30 October 2009	international travel & commerce		http://www.who.int/csr/don/2009_10_30a/en/
Legionnaires'	Netherlands	18 March 1999	international travel & commerce		http://www.who.int/csr/don/1999_03_18b/en/
Poliomyelitis	Namibia	07 June 2006	international travel & commerce		http://www.who.int/csr/don/2006_06_07/en/index.html
Poliomyelitis	Ethiopia	06 September 2005	international travel & commerce		http://www.who.int/csr/don/2005_09_06/en/
Poliomyelitis	Indonesia	06 June 2005	international travel & commerce		http://www.who.int/csr/don/2005_06_06/en/
Poliomyelitis	Kenya	02 March 2009	international travel & commerce		http://www.who.int/csr/don/2009_03_02a/en/
Poliomyelitis	Uganda	02 March 2009	international travel & commerce		http://www.who.int/csr/don/2009_03_02a/en/
Meningitis	DR Congo	02 February 2007	international travel & commerce		http://www.who.int/csr/don/2007_02_02/en/
Meningitis	Saudi Arabia (global spread)	11 April 2000	international travel & commerce		http://www.who.int/csr/resources/publications/meningitis/whocdscsrgar20021.pdf
Poliomyelitis	Sudan	02 March 2009	international travel & commerce		http://www.who.int/csr/don/2009_03_02a/en/
Shigellosis	Central African Republic	05 November 2003	international travel & commerce		http://www.who.int/csr/don/2003_11_05a/en/
Legionnaires'	Belgium	18 November 1999	international travel & commerce		http://www.who.int/csr/don/1999_11_18b/en/
Cholera	DR Congo	20 January 1997	war & famine		http://www.who.int/csr/don/1997_01_20/en/index.html
Legionnaires'	Norway	05 September 2001	breakdown of public health measures	sanitation & hygiene	http://www.who.int/csr/don/2001_09_05a/en/index.html
Enterovirus	Greece	26 April 2002	breakdown of public health measures	sanitation & hygiene	http://www.who.int/csr/don/2001_12_05/en/index.html
Ebola	Congo Republic	07 February 2003	bushmeat		http://www.who.int/csr/don/2003_02_07a/en/index.html
Poliomyelitis	Angola	01 July 2005	breakdown of public health measures	immunization coverage	http://www.who.int/csr/don/2005_07_01a/en/
Tularemia	Serbia	17 January 2002	food & agriculture industry changes		http://www.who.int/csr/don/2002_01_17/en/index.html
[illegible] Hemorrhagic	Congo Republic	12 June 2002	bushmeat		http://www.who.int/csr/don/2002_06_12e/en/index.html
Cholera	DR Congo	04 April 1996	breakdown of public health measures	sanitation & hygiene	http://www.who.int/csr/don/1996_04_04b/en/index.html and http://www.who.int/csr/don/1996_07_05b/en/index.html
Typhoid	Tajikistan	02 August 1996	climate & weather		http://www.who.int/csr/don/1996_08_02/en/
Plague	Zambia	31 January 1997	climate & weather		http://www.who.int/csr/don/1997_01_31b/en/
Avian Influenza	China	17 November 2005	food & agriculture industry changes		http://www.who.int/csr/don/2005_11_17/en/
Dengue	[illegible] (Guam; Cook Islands; Fiji; New Caledonia; Kiribati)	08 June 1998	climate & weather		http://www.who.int/csr/don/1998_06_08e/en/index.html
Dengue	Latin America (Brazil; Venezuela; Colombia)	08 June 1998	climate & weather		http://www.who.int/csr/don/1998_06_08e/en/index.html

Disease	Country	Date	General Driver	Driver Details	Link
Malaria	Jamaica	09 February 2007	climate & weather		http://www.who.int/csr/don/2007_02_09/en/index.html
Cholera	West Africa (Burkina Faso; Guinea; Guinea-Bissau; Liberia; Mali; Mauritania; Niger; Senegal; Benin)	26 August 2005	climate & weather		http://www.who.int/csr/don/2005_08_26/en/index.html
Buffalo Pox	India	11 April 1996	food & agriculture industry changes		http://www.who.int/csr/don/1996_04_11/en/
Cholera	DR Congo	15 November 1996	unspecified		http://www.who.int/csr/don/1996_11_15b/en/index.html
Ebola	Congo Republic	07 November 2003	unspecified		http://www.who.int/csr/don/2003_11_07/en/ and
Ebola	South Africa	18 November 1996	medical industry changes		http://www.who.int/csr/don/1996_11_18/en/
Cholera	Cameroon	15 June 2004	climate & weather		http://www.who.int/csr/don/2004_06_15/en/
Cholera	Zambia	27 January 1999	climate & weather		http://www.who.int/csr/don/1999_01_27/en/
SARS	China	22 April 2004	breakdown of public health measures	sanitation & hygiene	http://www.who.int/csr/don/2004_04_22/en/
Crimean-Congo Hemorrhagic Fever	Turkey	08 August 2006	breakdown of public health measures	sanitation & hygiene	http://www.who.int/csr/don/2006_08_08b/en/
Rift Valley Fever	Mauritania	10 November 1998	food & agriculture industry changes		http://www.who.int/csr/don/1998_11_10/en/
Rift Valley Fever	Yemen	26 September 2000	food & agriculture industry changes		http://www.who.int/csr/don/2000_09_26/en/
Crimean-Congo Hemorrhagic Fever	Pakistan	08 May 1998	food & agriculture industry changes		http://www.who.int/csr/don/1998_05_08b/en/
Crimean-Congo Hemorrhagic Fever	South Africa	05 November 1996	food & agriculture industry changes		http://www.who.int/csr/don/1996_11_05/en/
Japanese Encephalitis	India	13 September 2005	breakdown of public health measures	vector control	http://www.who.int/csr/don/2005_09_13a/en/
Crimean-Congo Hemorrhagic Fever	Afghanistan	08 May 1998	human susceptibility to infection		http://www.who.int/csr/don/1998_05_08a/en/
Cholera	Malaysia	20 May 1996	food & agriculture industry changes		http://www.who.int/csr/don/1996_05_20/en/
Cholera	Vietnam	22 April 2008	food & agriculture industry changes		http://www.who.int/csr/don/2008_04_22/en/
Cholera	Guinea-Bissau	24 September 2008	human susceptibility to infection		http://www.who.int/csr/don/2008_09_24/en/
Poliomyelitis	Iraq	24 September 1999	breakdown of public health measures	immunization coverage	http://www.who.int/csr/don/1999_09_24/en/
Rift Valley Fever	Kenya; Somalia	06 January 1998	human demographics & behavior		http://www.who.int/csr/don/1998_01_06b/en/index.html
Meningitis	DR Congo	28 January 1998	human demographics & behavior		http://www.who.int/csr/don/1998_01_28/en/
Cholera	Senegal	29 March 2005	human demographics & behavior		http://www.who.int/csr/don/2005_03_29/en/
Cholera	Rwanda	13 December 1996	human demographics & behavior		http://www.who.int/csr/don/1996_12_13a/en/
Cholera	Rwanda	19 May 1998	human demographics & behavior		http://www.who.int/csr/don/1998_05_19c/en/
Typhus	Burundi	06 May 1997	war & famine		http://www.who.int/csr/don/1997_05_06c/en/
Diphtheria	Afghanistan	29 August 2003	war & famine		http://www.who.int/csr/don/2003_08_29/en/
Hepatitis	Chad	19 August 2004	war & famine		http://www.who.int/csr/don/2004_08_19/en/
Leishmaniasis	Pakistan	16 January 2002	war & famine		http://www.who.int/csr/don/2002_01_16/en/
Meningitis	Chad	11 January 2005	war & famine		http://www.who.int/csr/don/2005_01_11a/en/index.html
Meningitis	Tanzania	23 August 2002	war & famine		http://www.who.int/csr/don/2002_08_23a/en/index.html
Shigellosis	Sudan	14 July 2004	war & famine		http://www.who.int/csr/don/2004_07_14/en/
Diarrheal Disease	Bangladesh	30 September 1998	breakdown of public health measures	sanitation & hygiene	http://www.who.int/bulletin/archives/78(9)1093.pdf
E. coli	Canada	30 May 2000	breakdown of public health measures	sanitation & hygiene	http://www.who.int/csr/don/2000_05_30/en/

Disease	Country	Date	General Driver	Driver Details	Link
Cholera	Zambia	27 October 1999	human demographics & behavior		http://www.who.int/csr/don/1999_10_27/en/
Rift Valley Fever	Kenya	26 December 2006	human demographics & behavior		http://www.who.int/csr/don/2006_12_26/en/
Yellow Fever	Brazil	14 January 2000	breakdown of public health measures	vector control	http://www.who.int/csr/don/2000_01_14/en/
Chikungunya	India	17 October 2006	breakdown of public health measures	vector control	http://www.who.int/csr/don/2006_10_17/en/
Crimean-Congo Hemorrhagic Fever	Russia	26 July 1999	breakdown of public health measures	vector control	http://www.who.int/csr/don/1999_07_26/en/
Cholera	Liberia	26 April 1996	war & famine		http://www.who.int/csr/don/1996_04_26/en/
Cholera	Tanzania	06 December 1996	war & famine		http://www.who.int/csr/don/1996_12_06c/en/
Undiagnosed Hemorrhagic Fever	Zimbabwe	14 May 1999	war & famine		http://www.who.int/csr/don/1999_05_14/en/
Meningitis	Angola	21 October 1999	war & famine		http://www.who.int/csr/don/1999_10_21/en/
Meningitis	DR Congo	22 December 1998	war & famine		http://www.who.int/csr/don/1998_12_22b/en/
Cholera	DR Congo	15 April 1997	unspecified		http://www.who.int/csr/don/1997_04_15b/en/index.html
Cholera	West Africa (Ghana; Sierra Leone)	14 October 1998	unspecified		http://www.who.int/csr/don/1998_10_14/en/index.html
Tularemia	Yugoslavia	21 April 2000	unspecified		http://www.who.int/csr/don/2000_04_21a/en/index.html
Arenavirus	Zambia; South Africa	10 October 2008	unspecified		http://www.who.int/csr/don/2008_10_13/en/index.html
SARS	China	28 December 2003	unspecified		http://www.who.int/csr/don/2003_12_28/en/index.html
Legionnaires'	France	13 August 1998	unspecified		http://www.who.int/csr/don/1998_08_13/en/index.html
Cholera	Ecuador	29 February 1996	human susceptibility to infection		http://www.who.int/csr/don/1996_02_29a/en/
Ebola	Gabon; Congo Republic	05 December 2001	unspecified		http://www.who.int/csr/don/2001_12_05/en/index.html
Yellow Fever	Liberia	25 February 2004	human demographics & behavior		http://www.who.int/mediacentre/news/releases/2004/pr16/en/index.html
Undiagnosed Hemorrhagic Fever	Gabon	24 June 2002	unspecified		http://www.who.int/csr/don/2002_06_24e/en/index.html
Crimean-Congo Hemorrhagic Fever	Yugoslavia	08 June 2001	unspecified		http://www.who.int/csr/don/2001_06_08e/en/index.html and http://www.who.int/csr/don/2001_06_29e/en/index.html
