# Supplementary material for: Preventing Pandemics Via International Development: A Systems Approach
Source: PLoS Med. 2012 Dec 11;9(12):e1001354. doi: 10.1371/journal.pmed.1001354 (PMC3519898; doi:10.1371/journal.pmed.1001354)
Supplement: Table S3 — Number of outbreaks by driver as used in Figure 2 . (PDF) [file pmed.1001354.s003.pdf]

Supplementary Table S3: Number of outbreaks by driver, used in Figure 2.

**Rank**	**Driver**	**Subdriver Count**	**Total Count**	**Percent**
1	*breakdown of public health measures*		157	0.40
	(sanitation & hygiene)	93		
	(immunization coverage	48		
	(vector control)	16		
2	*food & agriculture industry changes*		51	0.13
3	*climate & weather*		42	0.11
4	*international travel & commerce*		37	0.09
5	*unspecified*		36	0.09
6	*human demographics & behavior*		32	0.08
7	*war & famine*		20	0.05
8	*bushmeat*		12	0.03
9	*other*		10	0.03
	(human susceptibility to infection)	5		
	(land use changes)	4		
	(medical industry changes)	1		
		TOTAL EVENTS	*397*	
