# Supplementary material for: Preventing Pandemics Via International Development: A Systems Approach
Source: PLoS Med. 2012 Dec 11;9(12):e1001354. doi: 10.1371/journal.pmed.1001354 (PMC3519898; doi:10.1371/journal.pmed.1001354)
Supplement: Text S1 — Supplemental online material. (DOC) [file pmed.1001354.s004.doc]

**Text S1**

**Supplemental Online Material**

*Preventing pandemics via international development: A systems approach*

Tiffany L. Bogich, Rumi Chunara, David Scales, Emily Chan, Laura Pinheiro, Aleksei Chmura, Dennis Carroll, Peter Daszak and John S. Brownstein*

**WHO Outbreak Data**

Our dataset of 397 outbreaks from 1996 to 2009 is a subset of outbreaks reported in DON reports, according to Figure 6 in Chan *et al*. . See in particular the supplemental online material provided by Chan *et al.* for a detailed explanation of inclusion/exclusion criteria for outbreak reports. Unlike recent analyses , this dataset was selected to be infectious disease focused, concern outbreaks with multiple cases, include repeated outbreaks of pathogens, and include outbreaks that came to light through informal channels (e.g. epi-intelligence), which are not necessarily reported in the peer-reviewed literature .

Outbreaks used in the analysis are listed in Table S1 and the full reports for each event can be found online at <http://www.who.int/csr/don/en> or using the link provided with each event in the outbreak dataset in Table S1. For outbreaks with multiple reports (listed as ‘Update’ in the WHO DON database), the date from the original report is recorded, but all related reports were searched for driver data. Country of outbreak origin, date, disease, and the main factor driving the initial emergence were recorded, along with the text from the report used to make the driver assessment and a link to the original report. Driver identification methods are described in the next section.

Admittedly, the outbreaks in our dataset are not exhaustive. They are acute public health events officially recognized by the WHO as posing a potential international threat. As our data only covers outbreaks of diseases reported by WHO, it does not represent the complete burden of disease, encompassing both chronic and infectious diseases, emerging and endemic. Note that outbreaks of antibiotic resistant strains of diseases and historical outbreaks of disease (e.g. HIV/AIDS) are not reported in DON. Further, Chan *et al*. excluded from the dataset outbreaks of diseases such as foodborne, endemic, or seasonal infections that have lower pandemic potential.

**Identifying Drivers**

We identified the proximate driver implicated in the WHO DON reports for each of the 397 outbreaks through a manual text search of every outbreak report related to a single event. We limited driver categories to those identified by the Institute of Medicine . When summarizing data, drivers with fewer than 10 outbreaks were grouped in an ‘Other’ category (Table S3). The driving factors of emergence were first classified by the Institute of Medicine in 1992 and revised in 2003 and include 13 factors (Human demographics and behavior, Technology and industry, Economic development and land use, International travel and commerce, Microbial adaptation to change, Breakdown of public health measures, Human susceptibility to infection, Climate and weather, Changing ecosystems, Poverty and social inequity, War and famine, Lack of political will, and Intent to harm). We reclassified ‘Economic development and land use’ and ‘Technology and industry’ to form more descriptive categories: ‘Food & Agricultural industry changes’, ‘Medical industry changes’, ‘Land use changes’ and ‘Bushmeat’ according to . Further, ‘Poverty and social inequity’ and ‘Changing ecosystems’ were excluded, as they can be captured in other, more specific driving categories such as land use change, breakdown of public health services, bushmeat, and others. The final list of drivers assessed included:

| **Driving Factors** | | **Description** |
| --- | --- | --- |
| 1 | Agricultural & Food industry changes | Agricultural intensification of crops or animal husbandry and all aspects of food production from manufacturing to marketing; global trade and travel related to food are included here |
| 2 | Breakdown of public Health measures | Breakdown in public health measures that have previously worked to prevent infections, including inadequate sanitation and hygiene (e.g. shortages of potable water), poor immunization coverage or lack of infrastructure to purchase and deliver vaccine, TB control, control of vector-borne and zoonotic disease, and antiquated public health laws |
| 3 | Bushmeat | Both hunting and consumption of wildlife, including tracking, capturing and handling involved in hunting |
| 4 | Climate and weather | Short- and long-term fluctuations in temperature and rainfall affecting pathogens, vectors and hosts |
| 5 | Human demographics and behavior | Demographic and behavioral factors that affect human transmission of disease, examples include migration, population growth, aging, urbanization, and high-risk behaviors |
| 6 | Human susceptibility to infection | Elements of human physiology that determine susceptibility, including genetic, physical, cellular and molecular defenses. Examples include malnutrition, impaired immunity, genetic polymorphisms |
| 7 | Intent to harm | Bioterrorism or threat of attack using biological agents, typically via aerosol dissemination |
| 8 | International travel and commerce | Movement of humans and animals and other goods, including volume and speed of air travel, cruise ships - does not include global travel and trade related to food |
| 9 | Lack of political will | Lack of global political commitment and general complacency toward infectious diseases |
| 10 | Land use changes | Changes in land use patterns due to anthropogenic activities, including encroachment, expansion of agricultural activities or urban areas, deforestation, dams and irrigation infrastructure, or reforestation |
| 11 | Medical industry changes | Advances in the medical industry such as blood transfusions, use of plastic catheters or artificial heart valves); other examples include blood product safety, organ, tissue and xeno-transplantation |
| 12 | Microbial adaptation and change | Antibiotic use and resistance |
| 13 | War and famine | Armed conflict, loss of food security, and handicapping of medical infrastructure due to conflict, including the displacement of humans to refugee camps, for example, during war times |
| 14 | Unspecified | Unable to assign driver |

Further, we evaluated subcategories of the driver ‘breakdown of public health measures’, according to the Institute of Medicine categories: 1) inadequate sanitation and hygiene, which includes shortages of potable water, lack of proper hygiene and poor sanitary conditions in general, 2) poor immunization coverage or a lack of infrastructure to purchase and deliver vaccine, and 3) deterioration of vector-borne and zoonotic disease control, as these are often the first programs to be cut from budgets . All driver classifications (for both general and subcategories, where applicable) according to the outbreak reports are included in Table S1, and summarized in Table S2.

Because events in WHO DON are, by nature, those events that have received further investigation by the international community, driving factors were frequently identified and, often the exact phrases used to define drivers by the IOM were also used in the text of the outbreak report. Otherwise drivers had to be inferred from a description of the causes of or underlying processes described in the event report. We recorded two levels of information in Table S1: 1) ‘general driver’ corresponds to the IOM drivers and aligns with the classification in Figure 2 of the manuscript, and 2) ‘driver details’ includes additional categories for breakdown of public health measures only, used in manuscript Figure 2. Driver information was coded by single entry and checked by two independent coders to reduce subjectivity in classification. When multiple factors were listed (e.g. vaccination and vector control) the one listed first or associated with the greatest number of cases, if this information was provided, was used to produce manuscript Figure 2. Finally, when a driving factor could not be attributed to the specific outbreak in question, we deferred to the description of action taken immediately following the outbreak and inferred a driver from this information. When no driver was specified or the driving factor still could not be clearly determined or inferred (e.g. disagreement between sources), the event was classified as ‘unspecified’.

**Data Limitations**

It should be noted that rare cases of novel pathogens emerging in humans for the first time may be more related to the macroscale drivers such as climate, land use, international travel and trade or agricultural intensification rather than breakdown of public health measures, as we found for the outbreaks examined here. This is due to the low probability nature of these events and the more dramatic change required to preempt their emergence. We have not examined pathogens and their diseases of this nature and instead focus only on acute public health events that have garnered international response.

While uncertainty and biases in reporting must be considered, they exist in the direction of both developed and developing countries. For example, the database does not include outbreaks of drug-resistant microbes or foodborne infections, and therefore likely skews away from developed countries. Similarly, the focus of the WHO GAR tends to be on outbreaks in countries that need or request support, which most likely skews in the direction of developing countries. At the same time, it is widely acknowledged that lack of political will, economic capacity and healthcare infrastructure in developing countries mean that they likely underreport diseases of international concern, relative to developed countries . Scientifically, there is an urgent need for a true background measure of reporting and the burden of unidentified outbreaks globally, within which to frame investigations such as the one presented here. Local-scale efforts to measure the complete burden of disease have already begun , but are far from complete.

**Supplemental References:**

1. E. H. Chan *et al.*, Global capacity for emerging infectious disease detection. *Proceedings of the National Academy of Sciences* **107**, 21701 (December 14, 2010, 2010).

2. K. E. Jones *et al.*, Global trends in emerging infectious diseases. *Nature* **451**, 990 (2008).

3. J. Lederberg, R. E. Shope, S. C. J. Oakes, *Emerging Infections: Microbial Threats to Health in the United States*. (Institute of Medicine, National Academy Press, Washington D.C., 1992).

4. M. S. Smolinski, M. A. Hamburg, J. Lederberg, *Microbial Threats to Health: Emergence, Detection, and Response*. (The National Academies Press, Washington D.C., 2003), pp. 398.

5. N. Homaira *et al.*, Cluster of Nipah virus infection, Kushtia District, Bangladesh, 2007. *PLoS ONE* **5**, e13570 (2010 Oct, 2010).

6. G. T. Keusch, M. Pappaioanou, M. C. Gonzalez, K. A. Scott, P. Tsai, Eds., *Sustaining Global Surveillance and Response to Emerging Zoonotic Diseases*, (The National Academies Press, Washington, D.C., 2009).

7. V. T. Le *et al.*, Viral Etiology of Encephalitis in Children in Southern Vietnam: Results of a One-Year Prospective Descriptive Study. *Plos Neglect. Trop. Dis.* **4**, (Oct, 2010).

8. S. Luby *et al.*, Recurrent Nipah virus outbreaks in Bangladesh, 2001-2007. *American Journal of Tropical Medicine and Hygiene* **77**, 273 (Nov, 2007).
